# Pro-Angiogenic and Wound-Healing Potential of Bioactive Polysaccharides Extracted from Moroccan Algae *Osmundea pinnatifida*

**DOI:** 10.3390/life15101564

**Published:** 2025-10-07

**Authors:** Zakaria Boujhoud, Malek Eleroui, Amal Feki, Hajer Ben Saad, Marwa Kraiem, Ibtissam Youlyouz Marfak, Sanah Essayagh, Said Hilali, Riadh Badraoui, Hatem Kallel, Jean Marc Pujo, Ibtissem Ben Amara, Abderraouf Hilali

**Affiliations:** 1 Laboratory of Health Sciences and Technologies, Higher Institute of Health Sciences, Hassan First University of Settat, Settat 26000, Morocco; z.boujhoud@uhp.ac.ma (Z.B.); ibtissam.marfak@uhp.ac.ma (I.Y.M.); abderraouf.hilali@uhp.ac.ma (A.H.); 2 Laboratory of Medicinal and Environment Chemistry, Higher Institute of Biotechnology, University of Sfax, Sfax 3000, Tunisia; aroui.malek@gmail.com (M.E.); hajer.ben.saad@hotmail.fr (H.B.S.);; 3 Higher Institute of Applied Studies in Humanities of Kef, University of Jendouba, Jendouba 8189, Tunisia; 4 Laboratory of Agrifood and Health, Faculty of Science and Technology, Hassan First University of Settat, Settat 26000, Morocco; essayagh@gmail.com (S.E.); said.hilali@uhp.ac.ma (S.H.); 5 Department of Biology, College of Science, University of Hail, Hail P.O. Box 2440, Saudi Arabia; riadh.badraoui@fmt.utm.tn; 6 Section of Histo-Emryology and Cytogenetics, Medicine Faculty of Tunis, University of Tunis El Manar, Rommana 1068, Tunisia; 7 Tropical Biome and Immunopathology CNRS UMR-9017, University of French Guiana, Inserm U 1019, Cayenne 97300, French Guiana; kallelhat@gmail.com; 8 Intensive Care Unit, Cayenne General Hospital, Cayenne 97300, French Guiana; 9 Emergency Department, Cayenne General Hospital, Cayenne 97300, French Guiana

**Keywords:** seaweed therapeutics, antioxidant activities, neovascularization, CAM assay, accelerated healing, molecular interactions

## Abstract

Various therapeutic approaches have been explored to speed up wound healing, with angiogenesis being a crucial factor in this process and skin repair. This study shows that a polysaccharide extracted from the red alga *Osmundea pinnatifida* (PSOP) can promote angiogenesis and accelerate healing. The structural properties of PSOP were investigated using various techniques, including scanning electron microscopy, X-ray diffraction, Fourier–transform infrared spectroscopy, ultraviolet–-visible spectroscopy, and high-performance liquid chromatography coupled with a refractive index detector. Additionally, the in vitro antioxidant activity of PSOP was evaluated using the reducing power assay, total antioxidant capacity measurement, and DPPH (2,2-diphenyl-1-picrylhydrazyl) free radical scavenging tests. The PSOP extract exhibited significant pro-angiogenic effects in the avian chorioallantoic membrane model. Furthermore, the efficacy of PSOP-based hydrogels for wound healing was assessed in vivo using an excision wound model in Wistar rats. The results indicated accelerated wound healing, increased collagen deposition, and enhanced tissue regeneration. Computational studies suggest that the observed wound healing and pro-angiogenic effects may be attributed to the affinity of the PSOP units for cyclooxygenase-2 and vascular endothelial growth factor. These findings support the potential use of PSOP as a bioactive agent in wound care.

## 1. Introduction

Wounds caused by surgery, burns, cuts, or severe traumatic lacerations require effective healing strategies. Rapid restoration of microvascular connectivity is essential to reduce ischemia and hypoxia, facilitating tissue repair and the regeneration processes [1]. Acute wound healing typically takes up to four weeks and involves four phases: hemostasis, inflammation, proliferation, and remodeling [2]. Impairments in angiogenesis represent one of the primary causes of delayed wound healing, as insufficient vascularization leads to excessive inflammation and deprives the damaged tissue of oxygen and nutrients essential for regeneration [3]. Disruptions in these processes may result in chronic wounds, such as pressure sores, diabetic ulcers, and vascular wounds, characterized by persistent inflammation, resistance to therapies, and insensitivity to healing signals. These conditions significantly increase treatment costs and contribute to elevated morbidity and mortality rates [4,5].

Bioactive compounds from marine algae have been extensively studied for their potential therapeutic applications across various diseases. Algal polysaccharides, in particular, have attracted significant attention due to their diverse biological activities, including anti-inflammatory, anticoagulant, antitumor, antiviral, and antioxidant properties [6,7,8,9]. These compounds are biodegradable and non-toxic, possess adhesive properties, and have unique physical and chemical characteristics [10]. Recently, natural polysaccharides have gained interest as effective agents in skin therapeutics, particularly for their ability to promote cell regeneration and collagen synthesis, which enhances wound healing [11]. For example, polysaccharide-based creams derived from a macroalga have demonstrated remarkable healing properties attributed to their antioxidant and antibacterial effects [12]. Furthermore, algal polysaccharides have been shown to reduce ultraviolet radiation-induced skin damage by decreasing superoxide anion levels and enhancing endogenous antioxidant levels in the dorsal skin of mice [13]. Oxidative stress caused by the reactive oxygen species (ROS) induces cellular damage to DNA, proteins, and lipids, hindering tissue regeneration [14].

*Osmundea pinnatifida* (synonym *Laurencia pinnatifida*) is a red macroalga (Class: Florideophyceae; Order: Ceramiales; Family: Rhodomelaceae) widely distributed in marine environments. This alga is consumed as a food and dietary supplement due to its rich content of vitamins, proteins, amino acids, fiber, and fatty acids [15]. The biotechnological potential of *O. pinnatifida* includes antitumor, antiviral, antiprotozoal, antibacterial, and antifungal activities [16]. Aqueous and hydroethanolic extracts from this alga have antioxidant and anti-aging properties [17], while enzymatic extracts have antioxidant and anti-diabetic activities [18]. Despite this, the polysaccharide extracts from *O. pinnatifida* remain underexploited, and their biological applications have yet to be fully investigated.

The current study aimed to extract and characterize a polysaccharide from the red alga *Osmundea pinnatifida*, called PSOP. Structural characterization of PSOP was conducted using chemical and instrumental analyses, including high-performance liquid chromatography (HPLC-RID), UV–visible spectroscopy, FT-IR spectroscopy, X-ray diffraction (X-RD), and scanning electron microscopy (SEM). The molecular weight, total sugar content, sulfate levels, and protein content were all measured. The antioxidant activities of PSOP were assessed using three methods: DPPH free radical scavenging, total antioxidant capacity, and reducing power assays. The pro-angiogenic potential was evaluated in ovo using the chorioallantoic membrane (CAM) model in chicken embryos and further validated in vivo using an excision wound model in Wistar rats. In addition, a computational study was conducted to investigate the binding affinities and molecular interactions of PSOP with key targets, including cyclooxygenase 2 (COX-2) and vascular endothelial growth factor (VEGF).

Extensive evaluations of PSOP have demonstrated its significant antioxidant properties and ability to stimulate new blood vessel formation, creating an environment conducive to wound healing. Furthermore, this PSOP can maximize the use of natural extracts while minimizing the residues of chemical reagents. We hypothesize that this polysaccharide offers numerous advantages, including rich biological activity, low production costs, simple preparation, and high biocompatibility. These factors make it a promising candidate for treating chronic wounds, such as diabetic ulcers, and tissue regeneration, including skin replacement and soft tissue repair.

## 2. Material and Methods

### 2.1. Algae Collection and Identification

**Figure 1:**
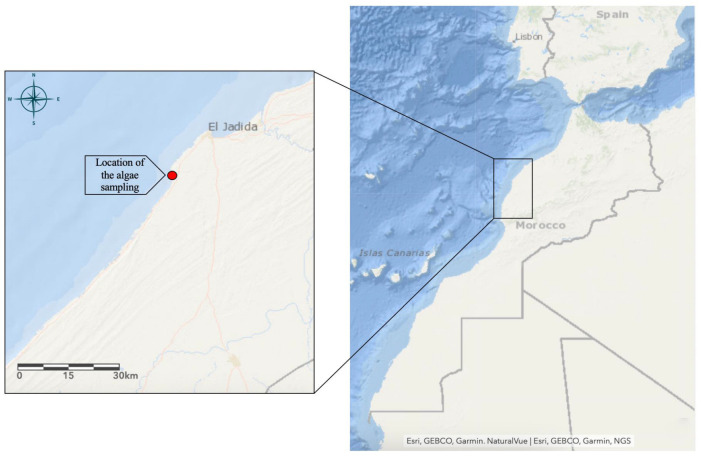
The location of the seaweed collection site was determined using ArcGIS version 10.7 (Sidi Bouzid coastal zone, El Jadida, Morocco).

*Osmundea pinnatifida* was collected from the intertidal zone of the rocky shore at Sidi Bouzid (El Jadida, Morocco; 33°13′ N, 8°55′ W) (Figure 1) between March and May 2023. Sampling occurred during low tide, with wave heights below 1 m. The algal samples were identified at the Marine Biotechnology and Environment Laboratory, Faculty of Science of Chouaib Doukkali University. Taxonomic identification was performed using an Olympus SZX12 stereomicroscope (Olympus, Tokyo, Japan) and a CX43 light microscope (Olympus, Tokyo, Japan). Resources used for identification included the Determination Key for Common Atlantic Coast Algae [19], the International Code of Nomenclature for Algae, Fungi, and Plants (ICN) [20], the Species Identification Guide [21], and the AlgaeBase [22].

Following identification, the samples were carefully washed, dried in the shade, ground into powder, and stored in amber glass bottles at 4 °C until further analysis.

### 2.2. Extraction of the Polysaccharide-Rich Osmundea Pinnatifida (PSOP)

The PSOP extraction process involved dissolving red algal pigments in 95% ethanol, following the methodology of Lui et al. [23]. A total of 100 g of dried algal powder was stirred in distilled water at 90 °C for 4 h. The aqueous extract obtained was filtered multiple times and evaporated under reduced vacuum pressure. The polysaccharide fraction was isolated by precipitation in 95% ethanol (3:1 *v*/*v*) at 4 °C for 24 h. Insoluble polysaccharides were collected by centrifugation (6000 rpm, 20 min, 4 °C) using a refrigerated centrifuge (Hettich Zentrifugen, Rotina 380R, Berline, Germany). The polysaccharides were then dissolved in distilled water and lyophilized using a freeze dryer (Christ Alpha 1–2 LD, Bioblock Scientific, Illkirch, France). The resulting extract was stored at 4 °C for further analysis.

The PSOP extraction yield (Y) was calculated using the following formula:

$$Y\%=\frac{Dry\;weight\;of\;extracted\;polysaccharide\;(g)}{Dry\;weight\;of\;Osmundea\;pinnatifida\;(g)}\times 100$$

### 2.3. Chemical Characterization

Total carbohydrate content was determined using the phenol–-sulfuric acid colorimetric method [24]. Protein concentration was measured by the Lowry method [25], and sulfate content was quantified using a turbidimetric method involving barium chloride and gelatin [26].

### 2.4. Polysaccharides Spectroscopic Analysis

#### 2.4.1. Ultraviolet Absorption Peak Detection

A 1% (*w*/*v*) solution of the polysaccharide was prepared in distilled water. The UV–visible absorption spectrum was recorded within a 200–800 nm wavelength range using a JENWAY/7315 spectrophotometer (UK) [27].

#### 2.4.2. Fourier Transform Infrared Spectrometry Analysis

FT-IR analysis of PSOP was conducted using a Nicolet FT-IR spectrometer equipped with an attenuated total reflection (ATR) attachment containing a diamond/ZnSe crystal. Spectra were recorded with a resolution of 4 cm^−1^ over 400–4000 cm^−1^ at room temperature (25 °C). Data analysis was performed using OPUS software version 7.0 (Bruker, Ettlingen, Germany) [28].

#### 2.4.3. Monosaccharide Analysis by High-Performance Liquid Chromatography (HPLC-RID)

Monosaccharides were analyzed using a high-performance liquid chromatography system (LC1260 Infinity II, Agilent technologies, Santa Clara, CA, USA) with a refractive index detector (RID). The sample was hydrolyzed in 2 M trifluoroacetic acid at 110 °C for 3 h, followed by methanol washing to remove residual acid. The hydrolyzed product was reduced using sodium tetrahydruroborate and then acetylated with pyridine and acetic anhydride at 40 °C for 2 h. The acetylated sample was filtered and injected into the system equipped with an Aminex HPX-87C column (Bio-Rad Laboratories, Hercules, CA, USA) at 60 °C, with 0.001 N H_2_SO_4_ as the mobile phase at a flow rate of 0.4 mL/min [29].

#### 2.4.4. The Average Molecular Weight

The average molecular weight (Mw) of PSOP was determined by high-performance gel permeation chromatography (HPGPC) (Allances 2695, Waters, Milford, MA, USA) using a refractive index detector. The analysis employed a Zorbax PSM 300 column (6.2 × 250 mm) with bidistilled water as the mobile phase, maintained at a flow rate of 0.8 mL/min and a temperature of 30 °C. Molecular weight calibration was performed using dextran standards with various molecular weights, following the method described by Bayar et al. [30].

#### 2.4.5. Scanning Electron Microscopy

Surface mapping, microstructure, and morphology of PSOP were analyzed using cryogenic scanning electron microscopy (JSM-IT500HR, Jeol, Tokyo, Japan). The samples were coated with a thin layer of gold to enhance conductivity before imaging.

#### 2.4.6. X-Ray Diffraction

The crystallinity of PSOP was assessed using X-ray diffraction (D2 PHASER, Bruker, Germany). The analysis was conducted at room temperature over a 2θ range of 10–80°, with a step size of 0.02° and a counting time of 5 s per step.

### 2.5. Antioxidant Activity of Polysaccharide

#### 2.5.1. DPPH Free Radical Scavenging Assay

The DPPH free radical scavenging activity of PSOP was assessed using the method previously described [31]. Different concentrations of PSOP (1–10 mg/mL) were incubated with 0.02% DPPH in ethanol. Absorbance was measured at 517 nm, and butylated hydroxyanisole (BHA) was used as the standard. The scavenging capacity was calculated with the following formula:

$$\%\;DPPH\;scavenging=\left[\frac{AC+AS-AB}{AC}\right]\times 100$$

where AC, AB, and AS correspond to the absorbance of the control, blank, and sample, respectively.

#### 2.5.2. Total Antioxidant Capacity

The total antioxidant capacity (TAC) of PSOP was measured according to the established protocol [32]. The reaction mixture was incubated, and absorbance was recorded at 695 nm. BHA served as the standard for this assay.

#### 2.5.3. Reducing Power Assay

The ferric reducing power of PSOP was assessed as described by Yıldırım et al. [33]. The reaction mixture was prepared, and absorbance was measured at 700 nm. BHA was used as the standard.

### 2.6. Evaluation of Angiogenic Activity

The angiogenic activity of PSOP was evaluated in ovo using the chorioallantoic membrane (CAM) assay with slight modifications based on the method described by Namvar et al. [34]. The experiment presented in this study does not require approval from the ethics committee [35].

Fertilized chicken eggs were sourced from Alf Sahel (Had Soualem, Morocco). The eggs were first cleaned with 10% betadine to ensure sterility and incubated at 37 °C with 55–65% relative humidity in an automatic egg incubator. The eggs were manually rotated three times a day to ensure proper embryonic development.

On day 3 of incubation, 3 mL of albumin was carefully extracted from each egg using a sterile syringe with a 20-gauge needle, creating space between the embryo and the eggshell. The eggs were returned to the incubator under the same conditions. On day 9, a small hole was drilled into the blunt end of the eggshell, and a window approximately 1 cm^2^ in size was created using sterile surgical scissors under a laminar flow hood. Part of the yolk membrane was carefully removed to avoid damaging the underlying CAM.

The eggs were divided into six experimental groups (n = 6 per group):

- Group 1 (Control): Sterile distilled water was applied.
- Group 2 (Negative Control): Diclofenac (DIC) at 50 μg/egg.
- Group 3 (Positive Control): Choriogonadotropin (CG) at 30 μg/egg.
- Group 4: PSOP at 25 μg/egg.
- Group 5: PSOP at 50 μg/egg.
- Group 6: PSOP at 100 μg/egg.

A volume of 200 μL of the treatment solutions was carefully applied to the surface of the CAM. The eggs were sealed with sterile transparent adhesive tape to prevent contamination and returned to the incubator for three additional days. On day 12 of incubation, the CAM was photographed using a stereomicroscope (Steroblue, EUROMEX, Hamburg, Germany), capturing detailed images of the neovascularized regions.

### 2.7. Quantitative Analysis of Vascular Network

Photomicrographs of the vascular network in the CAM were analyzed using ImageJ software (version 1.54, National Institutes of Health, Bethesda, MD, USA) and AngioTool software (version 0.6a, National Cancer Institute, Bethesda, MD, USA). Regions of interest (500 mm^2^) were selected from the images to quantify the topology of the vascular network.

The images were first converted to grayscale, and the “invert” function was applied using the ImageJ plugin under the “edit” menu. Background extraction was performed with a fixed value of 50 pixels using the “process” plugin. The processed images were then saved and imported into AngioTool software. Quantitative parameters were calculated to evaluate the angiogenic response, including vessel area, vessel length, vessel diameter, number of junctions, and lacunarity, representing the avascular space.

### 2.8. In Vivo Evaluation of Wound-Healing Activity in Rats

#### 2.8.1. Animals

Twenty-four healthy adult male Wistar rats weighing 150–200 g were procured from the Pasteur Institute of Tunis, Tunisia. The animals were housed in individually ventilated cages under controlled environmental conditions, including a temperature of 22–24 °C, a 12 h light/dark cycle, and unrestricted access to food and water.

The experimental protocol followed Directive 2010/63/EU on animal protection used for scientific purposes. The Higher Institute of Biotechnology at the University of Sfax, Tunisia, granted ethical approval (Protocol No. 09.0010/22, dated 25 January 2023).

#### 2.8.2. Experimental Protocol

An excision wound model was employed to evaluate wound healing. The rats were divided into four groups (n = 6 per group):

- Group 1 (Control): Treated with sterile saline (0.9% NaCl).
- Group 2 (Reference Drug): Treated with Cytol Centella, a commercially available medicated cream.
- Group 3 (PSOP Hydrogel): Treated with PSOP hydrogel prepared by mixing lyophilized PSOP in 30% glycerol to achieve a final 15 mg/mL concentration.
- Group 4 (Vehicle): Treated with 30% glycerol.

After sterilizing the skin with 70% alcohol, the back of each rat was shaved, and a circular wound measuring approximately 150 mm^2^ (1.5 cm in diameter) was created using a surgical blade. The wounds were cleaned and treated every two days with the assigned solutions. Treatments were applied as a thin layer to cover the wound surface completely. On day 12, all animals were sacrificed using ether anesthesia, followed by decapitation. Tissue samples from the wound site were collected for hydroxyproline content determination and histological examination.

#### 2.8.3. Measurement of Wound Area and Burn Contraction Rate

Wound healing was assessed by photographing the wound surface on days 1, 5, 7, and 11. The wound area was measured using Autodesk AutoCAD software version 24.2, and the rate of wound contraction was calculated using the following formula:

$$Rate\;of\;wound\;contraction\;\left(\%\right)=\frac{Initial\;wound\;area-Wound\;area\;on\;specific\;day}{Initial\;wound\;area}\times 100$$

#### 2.8.4. Determination of Hydroxyproline Content

Hydroxyproline content, an indicator of collagen deposition, was quantified, following the method described by Edwards et al. [36]. Tissue samples were dried at 60 °C until constant weight was achieved, hydrolyzed in 6 N HCl at 120 °C for 3 h, and subjected to chloramines-T-oxidation. The colorimetric reaction with Ehrlich’s reagent was developed at 60 °C, and absorbance was measured at 557 nm. Results were expressed as mg of hydroxyproline per gram of dry tissue.

#### 2.8.5. Histological Examination

On the day of complete wound closure, tissue samples from the wound area were collected, fixed in 10% formalin, embedded in paraffin, sectioned, and stained with hematoxylin–-eosin. Histological analysis was performed using a light microscope (Olympus CX41, Tokyo, Japan) to assess tissue regeneration and collagen deposition.

### 2.9. Computational Modeling and Interactions Assay

Computational modeling was performed to investigate the binding affinities of PSOP components with cyclooxygenase 2 (COX-2, pdb id: 1cx2) and vascular endothelial growth factor (VEGF, pdb id: 2c7w). Protein structures were retrieved from the RCSB Protein Data Bank and prepared by removing water molecules and adding polar hydrogens and Kollman charges. CHARMM force fields were applied as described in [37,38,39]. Binding interactions, including hydrogen bonds, hydrophobic interactions, and van der Waals forces, were analyzed and documented.

### 2.10. Statistical Analysis

All data were expressed as means ± standard deviation. Statistical analysis was performed using one-way analysis of variance (ANOVA) in SPSS software version 17.0. Tukey’s post hoc test was used for pairwise comparisons. Differences were considered statistically significant at *p* < 0.05.

## 3. Results and Discussion

### 3.1. Chemical Characterization of PSOP

The extraction yield of PSOP was calculated to be 32.65%. This value is significantly higher than the yields reported by Arunkuma et al. [40], where crude polysaccharides isolated from *Laurencia papillosa* and *Laurencia obtusa* yielded approximately 4% and 6%, respectively. However, this yield is lower than that of other polysaccharides extracted from other algal species: *Ulva lactuca* (47.3%) [41] and *Monostroma latissimum* (40.4%) [42]. Polysaccharide yields vary depending on species, collection period, environmental conditions, extraction methods, and solvents used [43,44].

The total sugar content of PSOP was determined to be 88.12 ± 1.1%, which is notably higher than the reported contents for *Laurencia pedicularioides* and *Laurencia cruciata* (44.8% and 49.4%, respectively) [45]. Colorimetric tests revealed low levels of protein (0.18 ± 0.02%) and sulfate (0.69%) in the PSOP extract, highlighting the precision of the employed extraction method. Polysaccharides obtained through ethanol precipitation often contain contaminants, such as proteins, which require further purification via deproteinization [46]. However, it is well-documented that deproteinization processes can affect the carbohydrate yields, alter the monosaccharide composition, modify the structural parameters, and influence the bioactivity of polysaccharides [47]. Additionally, sulfate groups associated with polysaccharides play a critical role in various biological activities, including antitumor, anticoagulant, and immunomodulatory effects [48].

### 3.2. Spectroscopic Analysis and Monosaccharide Composition

**Figure 2:**
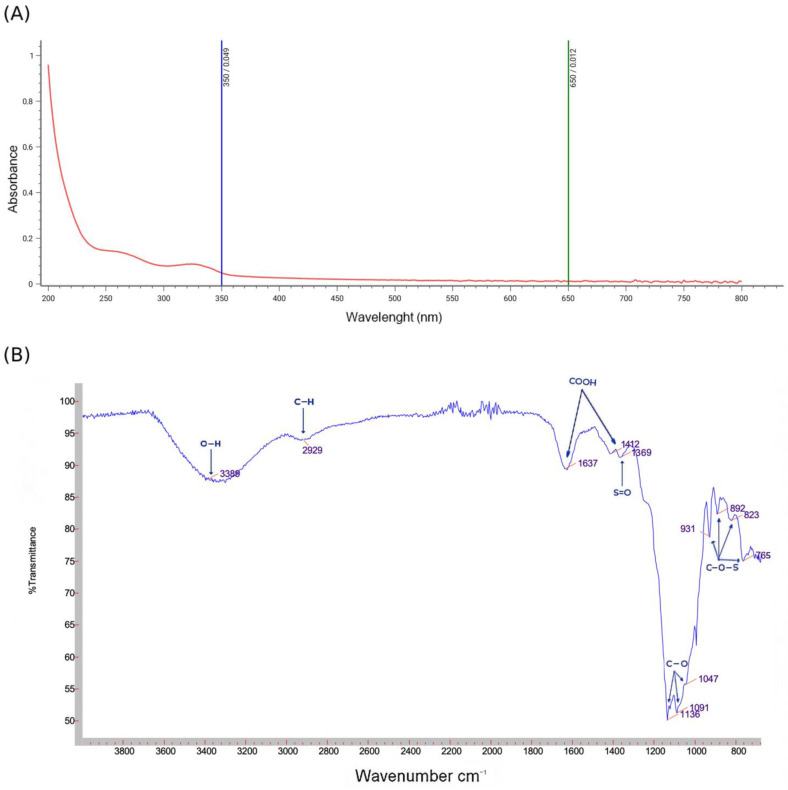
Preliminary characterization of PSOP. (**A**) UV–visible absorption spectrum of the PSOP; (**B**) Infrared spectrum of the PSOP.

Figure 2A illustrates the UV–visible spectra of PSOP, revealing a maximum absorption peak between 200 and 240 nm, confirming that the PSOP extract is a polysaccharide. These findings align with previous reports [49,50], which identified significant absorbance peaks in the 205–215 nm range. A weak peak was observed at 260–280 nm, indicating the presence of trace amounts of protein or nucleic acids covalently linked to PSOP [51]. Additionally, an absorption band near 300 nm was attributed to residual pigments.

FT-IR spectroscopy, widely used to identify the structural features of polymer blends and organic groups in polysaccharides, provided further insights into the composition of PSOP (Figure 2B). The FT-IR spectrum of PSOP exhibited characteristic absorption peaks for polysaccharides in the range of 700 to 4000 cm^−1^. A broad O-H stretching vibration peak at approximately 3389 cm^−1^ was attributed to intra- and intermolecular hydrogen bonds. A weak peak at 2929 cm^−1^ corresponded to C–H stretching vibrations of sugars [52], further confirming that PSOP is a polysaccharide compound. The peaks at 1637 and 1412 cm^−1^ were attributed to carboxylate bond stretching, indicating that PSOP is an acidic polysaccharide [53], potentially containing uronic acids. This observation was supported by the monosaccharide composition analysis. Polysaccharides’ hydroxyl and carboxyl groups enhance their biological activities, including antitumor and antioxidant effects [54]. A weak peak at 1369 cm^−1^ was assigned to the sulfate ester (S=O) groups [55]. The absorption bands between 1136 and 1047 cm^−1^ were associated with the C–O–C and C–O–H stretching vibrations, characteristic of the pyranose ring structure [56]. Finally, weak absorptions near 800 and 900 cm^−1^ were linked to C–O–S stretching vibrations [57], indicating the presence of α and β configurations within the polysaccharide.

**Figure 3:**
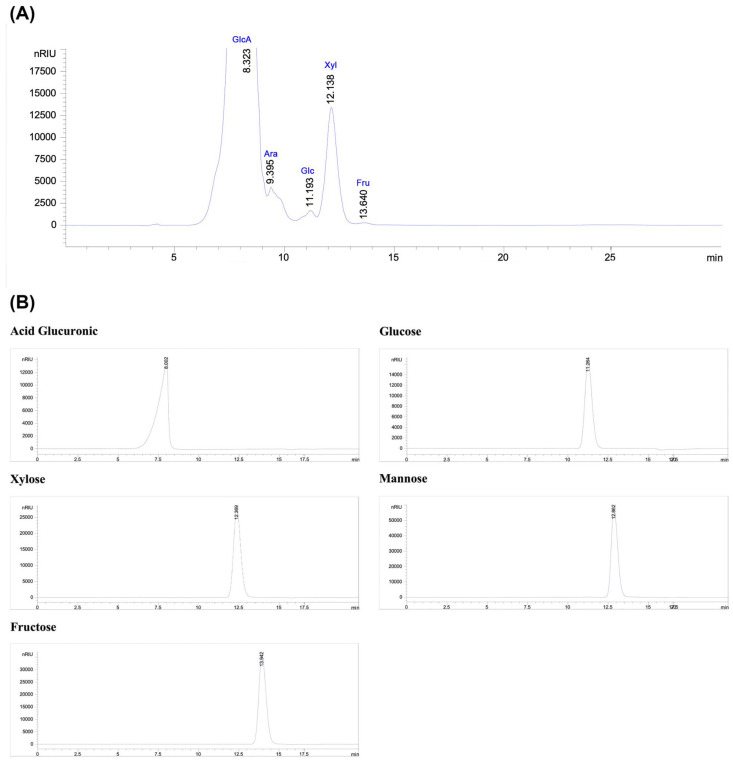
Preliminary characterization of PSOP. (**A**) Monosaccharide composition analysis by HPLC-RID; (**B**) Standards used for HPLC. GlcA: Acid Glucuronic; Ara: Arabinose; Man: Mannose; Glc: Glucose; Xyl: Xylose; and Fru: Fructose.

Figure 3A represents the HPLC-RID chromatogram of the monosaccharides detected in the PSOP. High-performance liquid chromatography with a refractive index detector identified the presence of glucuronic acid, arabinose, glucose, xylose, and fructose at the retention times of 8.32, 9.39, 11.19, 12.13, and 13.64 min, respectively, based on the elution times of monosaccharide standards (Figure 3B). The polysaccharides from red algae are known to consist of a heterogeneous combination of monosaccharides. For instance, the polysaccharide extracted enzymatically from *O. pinnatifida* contained glucuronic acid, galacturonic acid, galactose, mannose, arabinose, xylose, rhamnose, and fucose [58]. The polysaccharide composition of *Laurencia dendroidal* is predominantly galactose, with smaller amounts of xylose, mannose, and glucose [59]. However, both endogenous and exogenous factors can influence the monosaccharide composition of polysaccharides. Protocol variations, including water concentration, extraction temperature, and the reproductive cycle of the organism, are significant determinants [60,61]. The nature and proportion of monosaccharides are critical as they directly affect the bioactivity of polysaccharides, potentially conferring novel biological properties or enhancing existing ones [62].

**Figure 4:**
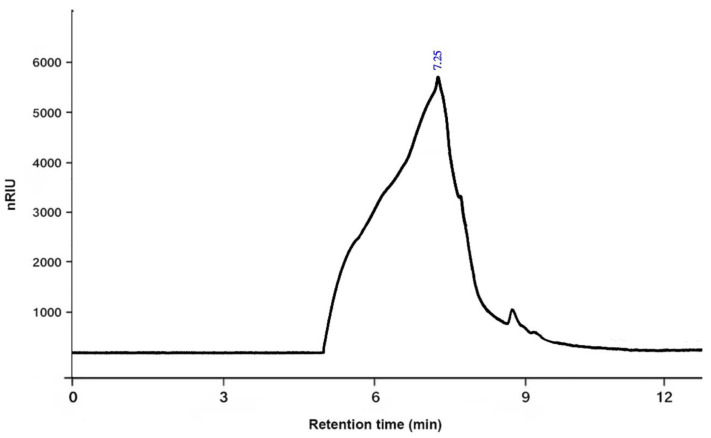
The average molecular weight of PSOP.

The molecular weight (Mw) of PSOP was determined using high-performance gel permeation chromatography (HPGPC). The analysis revealed a molecular weight of 146 kDa with a retention time of 7.25 min (Figure 4). The molecular weight of polysaccharides is a critical factor influencing their biological activities. A study conducted by Lee et al. [63] demonstrated that a low-molecular-weight polysaccharide (3 kDa), obtained through controlled degradation of a high-molecular-weight polysaccharide (2238 kDa), exhibited superior antioxidant activity. The low-molecular-weight polysaccharide more effectively stimulated the immune response in specific organs exposed to oxidative stress than the higher-molecular-weight polysaccharide. These findings highlight the potential for molecular weight optimization to enhance the bioactivity of polysaccharides.

**Figure 5:**
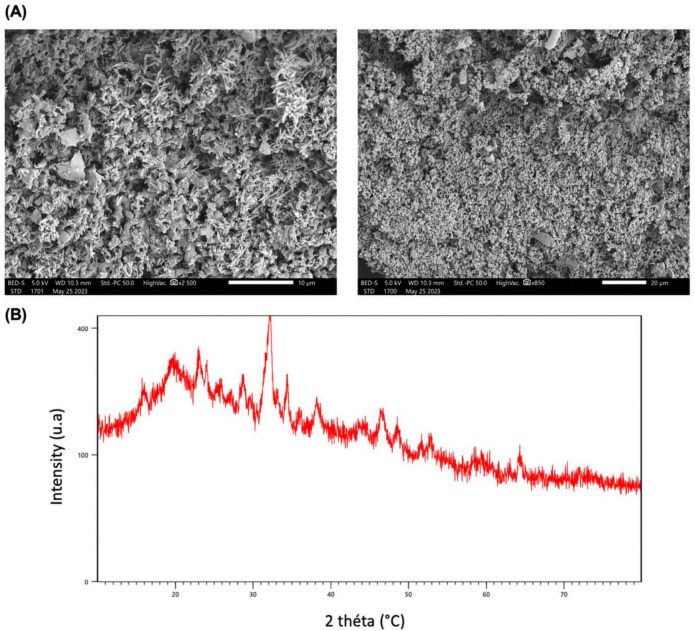
PSOP structure characterization. (**A**) SEM microstructure analysis of PSOP; (**B**) X-ray diffraction pattern.

Scanning electron microscopy (SEM) is a widely used technique to visualize the surface morphology of polysaccharides, providing insights into their structure, including shape, size, and porosity [64]. The SEM images of PSOP, presented in Figure 5A, reveal that under 10- and 20-fold magnification, PSOP exhibits a microstructure characterized by a network with numerous cavities and a highly developed porosity across the entire surface. Some heterogeneity in the structure was also observed, which enhances PSOP’s ability to supply oxygen and absorb exudates—features highly valuable in pharmacological and cosmeceutical applications [65].

X-ray diffraction (XRD) is commonly employed to determine the crystalline or amorphous structure [66]. As shown in Figure 5B, the XRD diffractogram of PSOP over a diffraction range of 0–80° displays a broad diffuse band with a dominant peak near 32° (2θ), accompanied by several smaller peaks. This pattern indicates that PSOP possesses a semi-crystalline structure embedded in an amorphous matrix. The degree of crystallinity in polysaccharides significantly impacts their properties, including mechanical strength, solubility, and swelling capacity [67]. For instance, semi-crystalline to crystalline polysaccharides demonstrate enhanced water retention capabilities, making them ideal for applications such as hydrogels, biofilms, nanofibers, membranes, and the medical field [68].

### 3.3. Antioxidant Activity of PSOP

**Figure 6:**
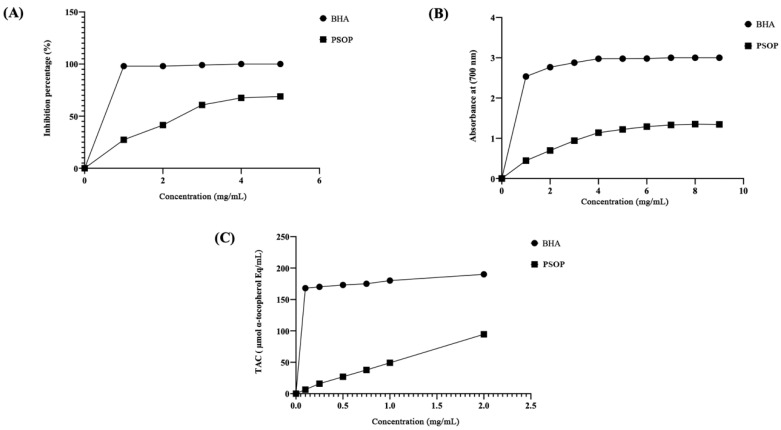
Antioxidant potentials of PSOP. (**A**) DPPH radical-scavenging activity; (**B**) Reducing power assay (RPA); and (**C**) Total antioxidant capacity (TAC).

Seaweeds are increasingly recognized as a natural source of antioxidant compounds [69]. These bioactive components are widely used in the cosmetics, pharmaceutical, and food industries to prevent oxidative damage and product degradation [70]. This study employed three assays to evaluate the antioxidant properties of PSOP extracted from *Osmundea pinnatifid*: DPPH free radical scavenging capacity, reduction power assay (RPA), and total antioxidant capacity (TAC). As illustrated in Figure 6A, the DPPH free radical scavenging activity of PSOP exhibited a concentration-dependent increase. At 5 mg/mL concentration, PSOP achieved a scavenging activity of approximately 68.9 %. However, butylated hydroxyanisole (BHA), a standard antioxidant, demonstrated significantly higher activity, reaching 97 % at a concentration of 0.5 mg/mL. The IC _50_ value for PSOP was 2.5 mg/mL, revealing that the scavenging capacity of PSOP was superior to that determined for *Halimeda tuna* (IC _50_ = 5 mg/mL) [71]. However, PSOP showed lower activity than polysaccharides isolated from *Hammada scoparia* (IC _50_ = 1.5 mg/mL) [50]. The antioxidant capacity of polysaccharides is closely associated with the presence of functional groups such as hydroxyls and carboxyl groups, as well as the sulfate content, which effectively scavenges free radicals like DPPH [72].

The reducing power assay, which measures the ability of a substance to act as an electron donor, was also conducted [73]. As shown in Figure 6B, the reducing power of PSOP increased with concentration, with maximum activity observed at 7 mg/mL (absorbance value of 1.33 at 700 nm). However, this value was lower than that of BHA, which reached 2.9 at 700 nm. The results indicated that the reducing power of PSOP appeared to be higher than that determined for *Codium tomentosum* (0.2 at OD 700 nm) at a concentration of 10 mg/mL [74]. The reducing power of polysaccharides is influenced by bioactive constituents such as uronic acids, glucuronic acids, and various monosaccharide units [75].

The total antioxidant capacity (TAC) of PSOP, assessed using the phosphorus–-molybdenum assay, is depicted in Figure 6C. The data indicate that PSOP displayed varying levels of activity that increased with concentration, although BHA consistently exhibited superior activity across all concentrations tested. For instance, at 1 mg/mL, BHA reduced molybdenum (Mo VI) to (Mo V) with an efficiency equivalent to 180 μmol/mL α-tocopherol compared to 49 μmol/mL α-tocopherol for PSOP at the same concentration. The polysaccharide extracted from *Asparagopsis armata* showed significant activity (130 μmol/mL α-tocopherol equivalents) for 1mg/mL [76]. According to Abdelhedi et al. [77], the TAC efficiency of polysaccharides may be attributed to their sulfate content.

### 3.4. Pro-Angiogenic Effect of PSOP

**Figure 7:**
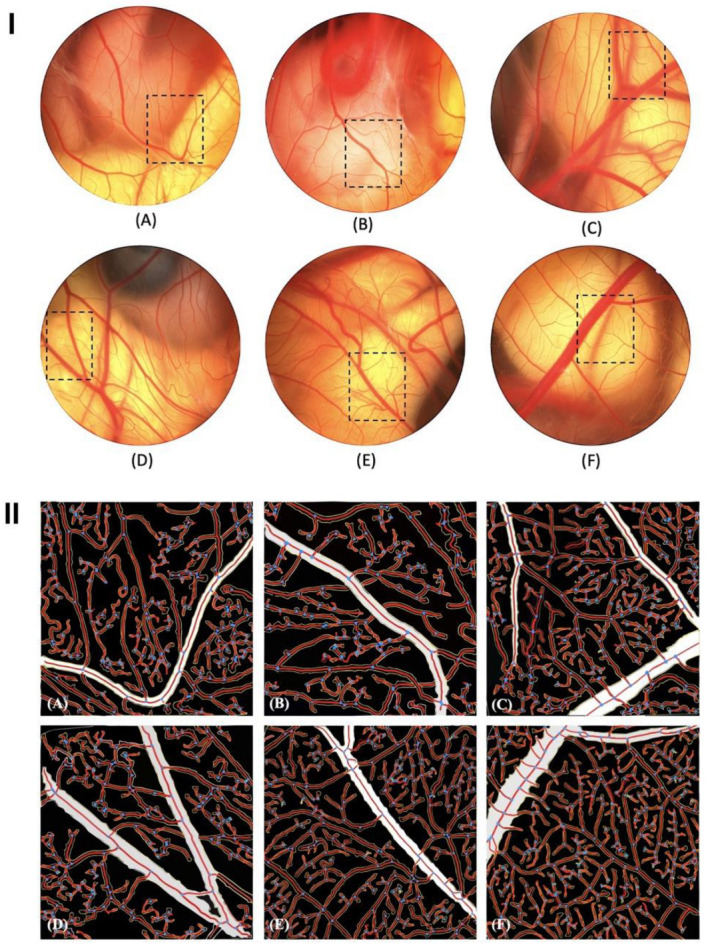
PSOP stimulated neovascularization of the chorioallantoic membrane in 12-day-old fertilized eggs. (**A**) Distilled water (control); (**B**) Diclofenac (50 μg/egg); (**C**) Choriogonadotropin (30 μg/egg); (**D**) PSOP (25 μg/egg); (**E**) PSOP (50 μg/egg); and (**F**) PSOP (100 μg/egg). (**I**): Original CAM images; (**II**): Resulting images after processing with AngioTool v 0.6a. The ring fields were photographed (magnification, ×10).

The chorioallantoic membrane (CAM) assay is widely used to evaluate various molecules’ angio-inhibitory and angio-stimulatory potential [78]. The results obtained in this study demonstrated that PSOP exhibits a clear and potent pro-angiogenic effect, as evidenced by a dose-dependent increase in the number of vessel branches. Figure 7I and Table 1 indicate that CAM treated with PSOP at concentrations of 25, 50, and 100 μg/egg showed significant neovascularization, with vessel development rates of 137.22 ± 3.7%, 200.66 ± 2.73%, and 250.66 ± 3.49%, respectively, compared to the untreated group, which was set at 100 % (*p* < 0.05). As a validation of the CAM model, diclofenac (DIC), an anti-angiogenic agent, significantly inhibited blood vessel formation, reducing neovascularization to 71.14 ± 3.97% compared to the untreated group (*p* < 0.05) [79]. Conversely, choriogonadotropin (CG), known for its pro-angiogenic properties, significantly enhanced blood vessel formation, reaching 168.36 ± 2.72% compared to the untreated group (*p* < 0.05) [80]. Angiogenesis is pivotal in forming new blood vessels, particularly in response to chronic or ischemic wounding [81]. This process is driven by hypoxia and inflammation, which recruit inflammatory cells and stimulate the release of growth factors that promote vascular development [82]. Among these factors, tumor necrosis factor-alpha (TNF-α) is a key pro-inflammatory cytokine that activates endothelial cells and enhances the production of vascular endothelial growth factor (VEGF) [83]. VEGF contributes to angiogenesis by promoting cell proliferation, stimulating the expression of specific genes, increasing nitric oxide production, and supporting cell survival [84,85]. Polysaccharides, including those derived from marine algae, have been shown to regulate the expression and activity of growth factors involved in angiogenesis, particularly VEGF [86,87]. The results of this study suggest that PSOP may influence angiogenesis by activating the inflammatory response and upregulating VEGF and TNF-α levels, thereby contributing to enhanced blood vessel formation.

### 3.5. Quantitative Analysis of Vascular Network

Figure 7II presents the vascular network images processed using the AngioTool software. In these images, vessel outlines are highlighted in yellow, vessels are marked in red, and junctions are indicated in blue. The analysis revealed that the density of blood vessels in the PSOP-treated groups was significantly higher compared to the control group, emphasizing the pro-angiogenic potential of PSOP. These findings further support the hypothesis that PSOP stimulates neovascularization, contributing to its potential as a pro-angiogenic agent.

**Figure 8:**
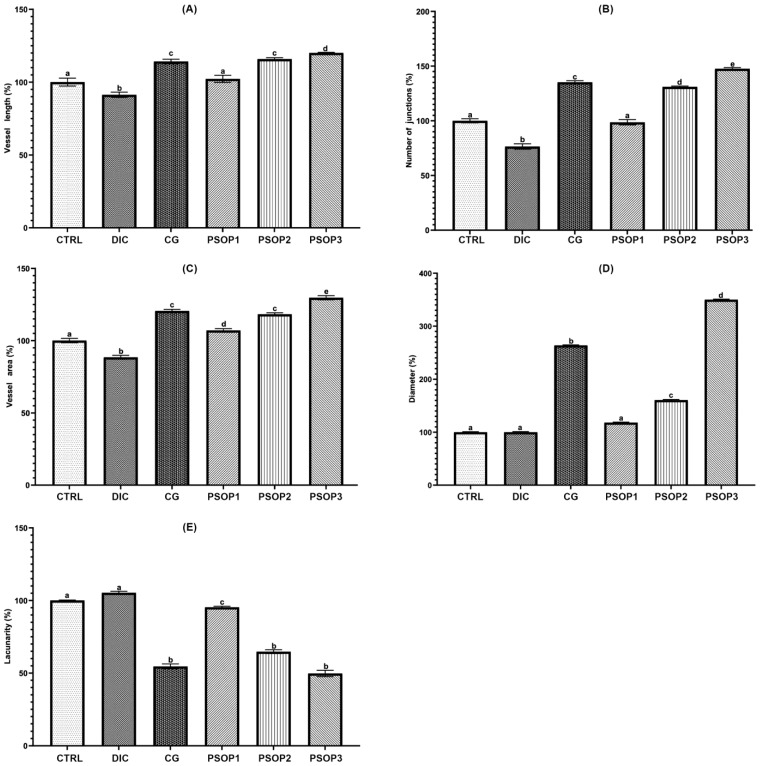
Effect of treatments on the density and morphology of the vascular network in the chorioallantoic membrane (CAM). (**A**) Vessel area, (**B**) Vessel length, (**C**) Vessel diameter, (**D**) Number of junctions, and (**E**) Lacunarity. Data are presented as percentages, and the control group is set to 100%. CTRL: Control; DIC: Diclofenac; CG: Choriogonadotropin; PSOP1: 25 μg/egg; PSOP2: 50 μg/egg; PSOP3: 100 μg/egg. The data represent mean ± SEM of six samples in each group. ^a–e^ indicate significant differences between different groups (*p* < 0.05).

Quantitative analysis of blood vessels, as depicted in Figure 8, demonstrates that PSOP extract at concentrations of 50 and 100 μg/egg, as well as choriogonadotropin (CG), significantly increased the vascular surface area, vessel length, vessel diameter, and the number of junctions compared to the control and the negative control diclofenac (DIC). However, treatment with PSOP extract at 25 μg/egg did not result in a significant difference compared to the control. Additionally, the lacunarity analysis revealed lower values in the PSOP-treated groups (50 and 100 μg/egg) and the positive control (CG) compared to the control and DIC-treated groups. Higher lacunarity values in the control and negative control groups indicate a greater frequency of empty spaces in the vascular network architecture. These findings suggest that the PSOP extract effectively enhances vascularization, promoting the formation of a more interconnected and denser vascular network. Improved vascularization is critical in wound healing, as it improves oxygen and nutrient delivery to the affected site and supports tissue regeneration, thus accelerating the healing process.

### 3.6. Evaluation of the Wound-Healing Activities of PSOP In Vivo

#### 3.6.1. Morphological Evaluation

**Figure 9:**
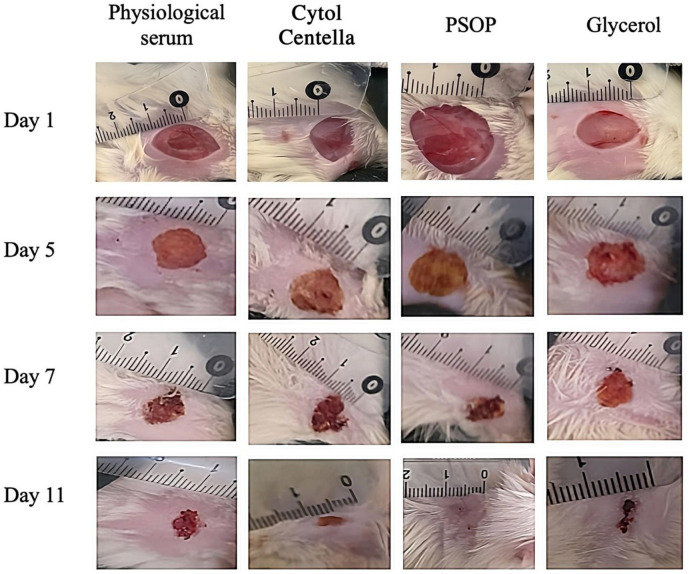
Representative photographs of the macroscopic appearance of 1.5 cm^2^ wounds excised on rats at days 1, 5, 7, and 11 of the group treated with physiological serum, glycerol, Cytol Centella, and PSOP hydrogel.

The progression of wound contraction was assessed by capturing images at various time intervals (days 1, 5, 7, and 11). As illustrated in Figure 9, the initial state of the wounds on day 1, immediately after induction and treatment application, exhibited a homogeneous appearance across all groups. The wounds displayed dark red coloration, well-defined margins, and visibly damaged tissue, confirming uniformity among groups at baseline. No discernible differences were observed between the groups at this stage, providing a benchmark for evaluating the efficacy of subsequent treatments. By day 5, wounds in the PSOP-treated and Cytol Centella-treated groups exhibited a brown discoloration, indicating crust formation due to the onset of blood coagulation healing. Both treatments showed substantial improvements in healing compared to the saline and glycerol-treated groups, characterized by significant wound size reduction and a less inflamed appearance.

In contrast, wounds in the untreated groups demonstrated delayed healing progress and a more pronounced inflammatory response. On day 7, saline and glycerol-treated wounds showed slower progression in tissue regeneration compared to those treated with PSOP and Cytol Centella. The latter treatments exhibited more advanced healing, suggesting accelerated tissue repair. By day 11, wounds treated with PSOP showed complete closure with no visible scarring, whereas open wounds and residual scab fragments persisted in the saline and glycerol-treated groups.

The morphological evaluation indicates that PSOP treatment accelerates healing and restores the skin’s structural integrity and coloration. Reactive oxygen species (ROS) are known contributors to delayed wound healing. Excessive ROS production, without an adequate antioxidant response, leads to the oxidative damage of cellular membranes, proteins, and DNA, resulting in prolonged inflammation and extracellular matrix degradation, which impedes wound healing [88]. Polysaccharides are recognized for neutralizing ROS, reducing oxidative stress by scavenging oxidizing free radicals, and preventing cellular and tissue damage [89]. These findings suggest that the antioxidant properties of PSOP mitigate oxidative stress, reduce wound stress, and promote faster and more effective healing.

#### 3.6.2. Assessment of Wound Area

**Figure 10:**
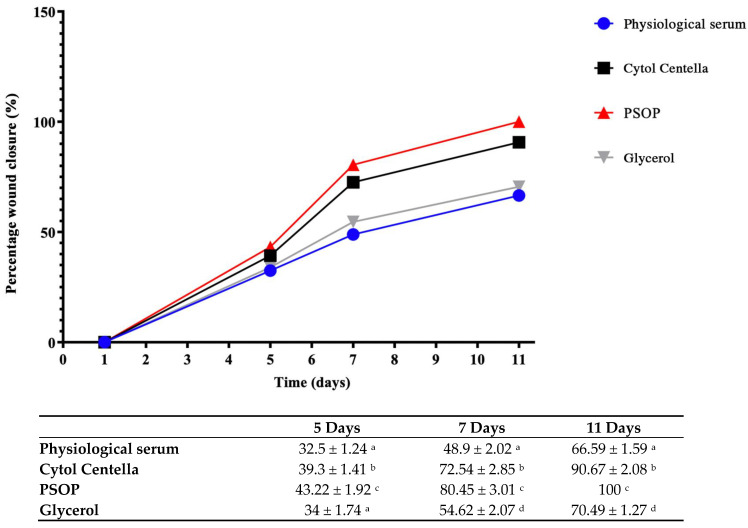
Percentage of wound area contraction in different groups of rats. Each value represents the mean ± SEM of results from 6 rats. ^a–d^ in the same column indicate significant differences (*p* < 0.05).

Epithelialization is a critical phase of wound healing during which epithelial cells migrate upwards to cover and repair the injured area [90]. The healing efficacy of the treatments was evaluated over the 11-day experimental period by regularly measuring the wound area. As shown in Figure 10, the groups treated with PSOP and Cytol Centella exhibited significantly enhanced healing compared to the untreated groups (glycerol and saline). By day 11, the PSOP-treated group achieved complete wound closure (100%), demonstrating optimal efficiency for tissue regeneration. The Cytol Centella group also showed substantial healing, achieving a wound reduction of 90.67 ± 0.27%, although slightly lower than that observed with PSOP.

In contrast, slower healing rates were observed in the glycerol and saline groups, with wound closure rates of 70.49 ± 1.09% and 66.59 ± 1.10 %, respectively. These results indicate that PSOP treatment promotes faster and more complete restoration of skin structure, highlighting its potential as an effective pro-healing agent. The epithelialization process is strongly influenced by wound conditions, particularly the moisture content of the wound environment. A moist environment fosters epidermal cell migration, accelerates re-epithelialization, and maintains high levels of proteinases and growth factors essential for optimal wound healing [91]. Polysaccharides from algae contribute significantly to maintaining a moist wound-healing environment due to their hydrophilic properties, biocompatibility, and moisture-retention capabilities [92].

#### 3.6.3. Hydroxyproline and Collagen Regeneration

**Figure 11:**
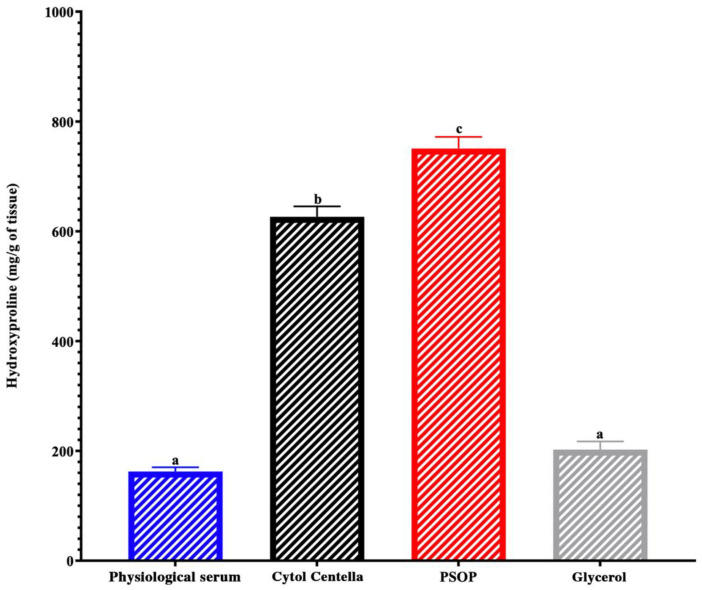
Hydroxyproline concentrations in biopsies from experimental rat wounds. Different letters on each bar indicate significant differences between treatments (*p* < 0.05).

To confirm the regeneration of skin tissues, we quantified hydroxyproline levels in wound area biopsies across all experimental groups. As shown in Figure 11, the PSOP-treated group demonstrated significantly higher hydroxyproline levels (750.81 ± 21 mg/g tissue) than the other groups. The levels were 162.53 ± 7.71 mg/g for the saline group, 202.45 ± 14.8 mg/g for the glycerol group, and 626.5 ± 18.95 mg/g for the Cytol Centella group, all with *p* < 0.05. Collagen, a major structural protein in connective tissue, is essential for wound healing. It facilitates tissue regeneration and restores the repaired skin’s elasticity, strength, and functionality [93]. Hydroxyproline, a collagen-specific amino acid, is a marker of collagen synthesis. Elevated hydroxyproline levels reflect increased collagen fiber formation, promoting cell proliferation and wound remodeling [94]. Hydroxyproline is produced through hydroxylation of proline, an enzymatic process mediated by prolyl hydroxylase, which requires cofactors such as oxygen and iron. This post-translational modification stabilizes the collagen’s triple helix structure, ensuring connective tissue strength and elasticity [95].

Polysaccharides have been shown to influence key biological pathways that promote collagen synthesis and angiogenesis. For instance, Zhang et al. [96] demonstrated that polysaccharides enhance the expression of interleukin-1 beta (IL-1β), which subsequently stimulates vascular endothelial growth factor (VEGF) production. This signaling cascade supports angiogenesis and collagen synthesis, which are critical for tissue regeneration and wound closure. Furthermore, the PI3K/Akt/mTOR signaling pathway, which regulates cell proliferation, survival, and collagen synthesis, is another mechanism influenced by polysaccharides [97]. Studies suggest that polysaccharides can activate these pathways, indirectly enhancing hydroxyproline production and contributing to collagen stability and tissue repair [98,99]. These findings and experimental data support the hypothesis that PSOP modulates these pathways, facilitating collagen synthesis and promoting effective skin tissue regeneration and wound healing.

#### 3.6.4. Histological Evaluation

**Figure 12:**
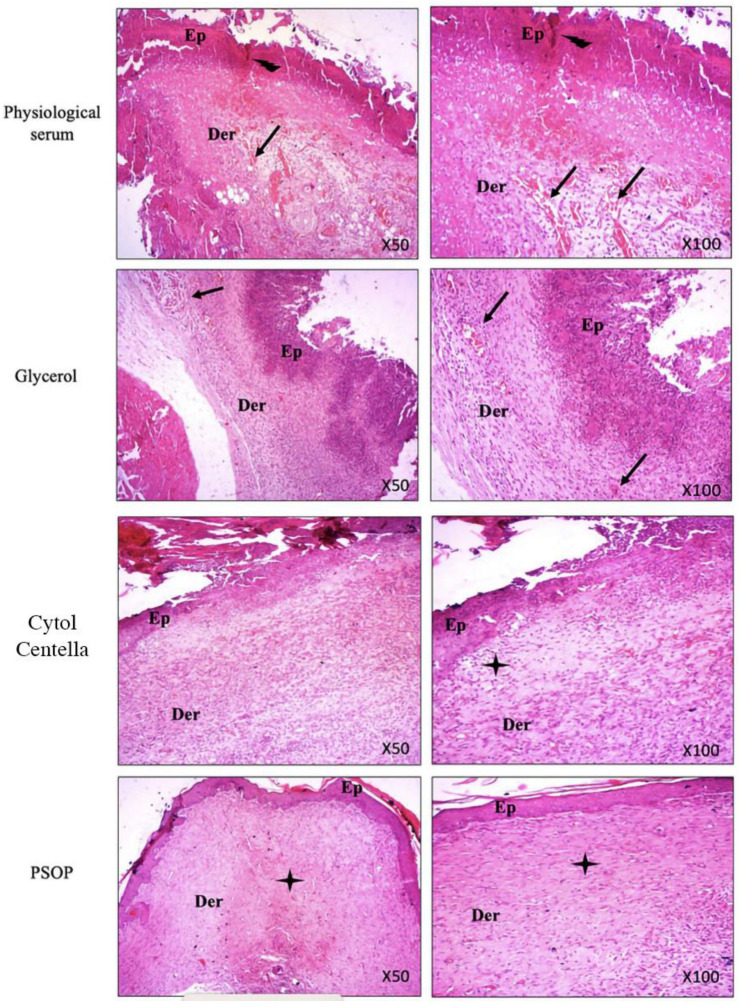
Representative microphotographs of epidermal and dermal wound structure on day 11 in rats treated with saline, glycerol, Cytol Centella, and PSOP hydrogel. Tissues were visualized at 50 and 100× magnification. Ep: epiderm and Der: derm. Arrows indicate the following: 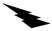 ulceration, 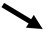 inflammatory infiltrate, 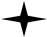 and fibers of collagen.

Re-epithelialization is a crucial phase in wound healing, involving the proliferation and migration of epithelial cells over the wound surface to form a protective layer over the regenerating tissue. Granulation tissue formation, neovascularization, and collagen deposition are fundamental mechanisms that facilitate cutaneous wound repair [100]. To assess the degree of wound healing, tissue sections from both treated and untreated groups were stained with hematoxylin–eosin and examined microscopically after the experimental period. The analysis focused on re-epithelialization, collagen and fibroblast formation, neovascularization, and the presence of inflammatory cells. As shown in Figure 12, microscopic images of tissue from the untreated groups (saline and glycerol) revealed persistent inflammatory infiltrate, necrosis in the epidermis and dermis, and low levels of collagen synthesis, indicative of incomplete repair and re-epithelialization. Conversely, tissue sections from the PSOP and Cytol Centella-treated groups demonstrated dense epithelization, robust neovascularization, and no inflammatory cells. These observations are consistent with earlier findings on hydroxyproline levels in wound biopsies.

In particular, histological sections from the PSOP-treated group showed complete healing, characterized by accelerated cell proliferation, enhanced protection against oxidative damage, and reduced inflammation. The findings also confirmed PSOP’s pro-angiogenic properties, likely mediated by stimulating VEGF expression, which promotes wound repair through multiple biological pathways.

### 3.7. Computational Findings

**Figure 13:**
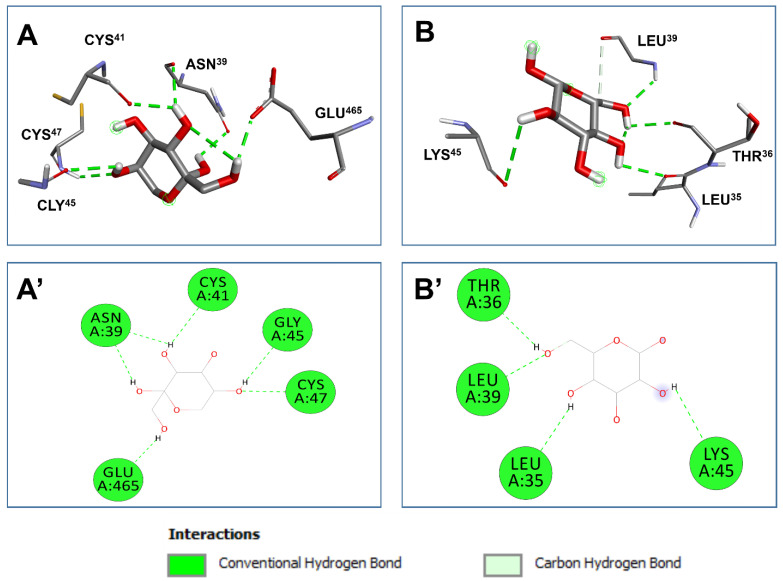
Three-dimensional illustration of the pocket regions (**A**,**B**) and resulted diagrams of interactions (**A’**,**B’**) of the monosaccharides identified from *O. pinnatifida* with the two targeted receptors: 1cx-2 and 2c7w for cyclooxygenase 2 (COX-2, (**A**)) and vascular endothelial growth factor (VEGF, (**B**)), respectively.

The monosaccharides identified in PSOP exhibited varying affinities to two targeted receptors, COX-2 and VEGF, as summarized in Table 2. All identified monosaccharides demonstrated negative binding affinities, suggesting their potential biological activity. The predicted binding affinities ranged between −5.6 and −6.4 kcal/mol for COX-2 and between −4.1 and −4.6 kcal/mol for VEGF. These variations are consistent with the influence of the three-dimensional chemical structures of the ligands [101,102]. The identified monosaccharides established acceptable molecular interactions with both receptors, forming four to six conventional hydrogen bonds, as detailed in Table 3. Key residues involved in these interactions include ASN^39^, CYS^47^, GLU^465^, CYS^41^, and GLY^45^, when fructose was complexed with COX-2 (Table 3 and Figure 13). For VEGF, glucose interacted twice with LEU^39^ and once with THR^36^, LEU^35^, and LYS^45^. PSOP monosaccharides were deeply embedded within the receptor pocket regions, with a proximity of up to 2.072 Å, indicating strong binding.

Deep embedding (<2.5 Å), as observed in this study, has been previously associated with enhanced bioactivities, such as antiproliferative, anti-inflammatory, and antimicrobial effects, and alleviation of toxicity [38,71,101,102]. The combination of binding affinities, deep embedding, and molecular interactions observed in PSOP components suggests that these chemicals’ pro-angiogenic and wound-healing effects are thermodynamically feasible. These predicted bioactivities are supported by the experimental results obtained in rat models. The findings reinforce the beneficial effects of natural-derived compounds, phytotherapy, and medicinal plants, including algae [71,101,103]. Computational modeling and interaction assays provide valuable insights into compounds’ drug-like properties and targeted proteins’ functional mechanisms. This study’s computational predictions elucidated the mechanistic pathways through which PSOP exerts its pro-angiogenic and wound-healing effects. The agreement between computational predictions and experimental results underscores the importance of integrating computational and experimental approaches in studying bioactive compounds [71,101,102].

## 4. Conclusions

This study provides a novel report on the antioxidant, wound healing, and pro-angiogenic properties of PSOP extracted from the red alga *Osmundea pinnatifida*. PSOP demonstrated significant antioxidant activities, as evidenced by various assays, including DPPH, TAC, and RPA. The findings also highlight PSOP’s pro-angiogenic potential, validated through the CAM assay, and its ability to accelerate wound healing in Wistar rats. Over an 11-day experimental period, PSOP treatment promoted angiogenesis, fibrotic tissue formation, and remodeling and reduced overall healing time in a circular excision wound model. Combined experimental and computational data suggest that PSOP could be utilized as a natural bioactive material for enhancing the healing of chronic wounds, such as diabetic ulcers and burns, or to promote vascularization during skin grafting procedures.

This study has several limitations that should be highlighted. Firstly, the molecular mechanisms underlying the pro-angiogenic and wound-healing effects were not thoroughly investigated. Furthermore, while the in vitro and in vivo tests yielded promising results, additional research, including genotoxicity analyses, is needed to verify the efficacy and safety of this extract in human models. Addressing these areas in future studies could enhance the relevance and applicability of these findings.

## Figures and Tables

**Table 1:** Percentage of blood vessels in CAM assay.

Groups	Control	Diclofenac (50 μg/egg)	Choriogonadotropin (30 μg/egg)	PSOP (25 μg/egg)	PSOP (50 μg/egg)	PSOP (100 μg/egg)
**Vessel Number (%)**	100 ^a^	71.14 ± 3.97 ^b^	168.36 ± 2.72 ^c^	137.22 ± 3.7 ^d^	200.66 ± 2.73 ^e^	250.66 ± 3.49 ^f^

Data are presented as percentages, and the control group is set to 100%. Values are expressed as means ± SD for 6 eggs in each group. ^a–f^ indicate significant differences between different groups (*p* < 0.05).

**Table 2:** Binding affinity of root mean square deviation of the monosaccharides identified from *O. pinnatifida* with the two targeted receptors: 1cx2 and 2c7w for cyclooxygenase 2 (COX-2) and vascular endothelial growth factor (VEGF), respectively.

Compound No.	COX-2 (1cx2)	VEGF (2c7w)
	Binding Affinity (kcal × mol^−1^)	
Arabinose	−5.7	−4.5
Fructose	−6.4	−4.5
Glucose	−6.3	−4.6
Glucoronic acid	−5.6	−4.4
Xylose	−6.1	−4.1
	RMSD (lower–upper)	
Arabinose	0.0–30.13	0.0–31.90
Fructose	0.0–30.82	0.0–9.08
Glucose	0.0–22.70	0.0–9.14
Glucoronic acid	0.0–41.78	0.0–14.83
Xylose	0.0−9.86	0.0–30.91

**Table 3:** Several conventional H-bonds, closest interacting residues, and distance to closest interacting residue (Å) of the monosaccharides identified from *O. pinnatifida* with the two targeted receptors: 1cx2 and 2c7w, for cyclooxygenase 2 (COX-2) and the vascular endothelial growth factor (VEGF), respectively.

Saccharide	No. of Conventional H-Bonds	Closest Interacting Residues		
		Interacting Residues	Closest residue (Distance, Å)	No. of Closest Interacting Residues
Cyclooxygenase 2 (COX-2)				
Fructose	6	CYS^47^, ASN^39^, GLU^465^, CYS^41^, GLY^45^	GLU^465^:OE1 (2.072)	5
Vascular Endothelial growth factor (VEGF)				
Glucose	4	LEU^39^, THR^36^, LEU^35^, LYS^45^	LEU^39^:HN (2.247)	4
Bold residues: amino acids interacting with conventional H-bond				

## Data Availability

The original contributions presented in the study are included in the article, further inquiries can be directed to the corresponding author.
